# The ELK3-DRP1 axis determines the chemosensitivity of triple-negative breast cancer cells to CDDP by regulating mitochondrial dynamics

**DOI:** 10.1038/s41420-023-01536-5

**Published:** 2023-07-08

**Authors:** Joo Dong Park, Hye Jung Jang, Seung Hee Choi, Gae Hoon Jo, Jin-Ho Choi, Sohyun Hwang, Wooram Park, Kyung-Soon Park

**Affiliations:** 1 Department of Biomedical Science, CHA University, Seongnam, Republic of Korea; 2 Department of Integrative Biotechnology, Sungkyunkwan University, Suwon, Republic of Korea

**Keywords:** Chemotherapy, Breast cancer

## Abstract

Triple-negative breast cancer (TNBC) is the most lethal form of breast cancer. TNBC patients have higher rates of metastasis and restricted therapy options. Although chemotherapy is the conventional treatment for TNBC, the frequent occurrence of chemoresistance significantly lowers the efficacy of treatment. Here, we demonstrated that ELK3, an oncogenic transcriptional repressor that is highly expressed in TNBC, determined the chemosensitivity of two representative TNBC cell lines (MDA-MB231 and Hs578T) to cisplatin (CDDP) by regulating mitochondrial dynamics. We observed that the knockdown of *ELK3* in MDA-MB231 and Hs578T rendered these cell lines more susceptible to the effects of CDDP. We further demonstrated that the chemosensitivity of TNBC cells was caused by the CDDP-mediated acceleration of mitochondrial fission, excessive mitochondrial reactive oxygen species production, and subsequent DNA damage. In addition, we identified *DNM1L*, a gene encoding the dynamin-related protein 1 (a major regulator of mitochondrial fission), as a direct downstream target of ELK3. Based on these results, we propose that the suppression of ELK3 expression could be used as a potential therapeutic strategy for overcoming the chemoresistance or inducing the chemosensitivity of TNBC.

## Introduction

Triple-negative breast cancer (TNBC) is an aggressive form of breast cancer, which lacks the three receptors that are present in more common types of breast cancer; namely, the estrogen receptor, the progesterone receptor, and the human epidermal growth factor receptor type 2. Patients with TNBC have higher rates of metastasis and worse overall survival than those with hormone-receptor-positive breast cancer [1, 2]. Due to a lack of available targeted therapies, chemotherapy is currently the primary systemic treatment option for TNBC patients. Platinum-based chemotherapy, including cisplatin (CDDP), has been widely used as a neoadjuvant treatment for TNBC [3]. Despite the fact that 20% of TNBC patients exhibit a pathologic complete response (pCR) to neoadjuvant chemotherapy, patients without a pCR are more likely to experience early recurrence and metastasis [4]. To achieve the desired therapeutic effect, it is therefore essential to better comprehend the molecular mechanism underlying the response of TNBC to chemotherapy.

Mitochondria are highly dynamic organelles that continuously undergo morphological alterations. These “mitochondrial dynamics” are necessary for mitochondrial quality control and function [5, 6]. Although mitochondria play critical roles in the chemotoxicity of cancer cells, the effect of mitochondrial dynamics on the therapeutic efficacy of chemotherapy is under debate. For instance, mitochondrial fission, followed by the mitophagy of dysfunctional daughter mitochondria, can contribute to cancer cell survival by eliminating irreparable mitochondrial (mt)DNA damage caused by chemotherapy [7]. Conversely, significantly higher levels of mitochondrial fusion have been observed in CDDP-resistant gynecological cancer cells, whereby the fused mitochondria support cancer cell survival by increasing ATP production [8]. Therefore, understanding how mitochondrial dynamics regulate the cellular response to chemotherapy is critical for overcoming chemoresistance in cancer therapy.

The erythroblast transformation specific (ETS)-domain-containing transcription factor, ELK3, belongs to the ternary complex factor subfamily. ELK3 typically functions as a transcriptional repressor. However, phosphorylation of ELK3 transforms it into a transcriptional activator [9, 10]. ELK3 is implicated in various biological processes such as neural cell development, angiogenesis, and tumorigenesis [11–13]. We have recently reported that ELK3 activity is also associated with mitochondrial dynamics [14]. *ELK3* knockdown in TNBC cells increased the expression of the mitochondrial fission adapter protein, MiD51, and consequently the rate of mitochondrial fission. This led to reactive oxygen species (ROS) accumulation and rendered TNBC cells more sensitive to natural killer cell-mediated cytotoxicity. Considering that mitochondria have a multitude of functions within eukaryotic cells, it is likely that the significance of the ELK3-mediated regulation of mitochondrial dynamics could extend beyond the immune response of cancer cells.

Here, we demonstrated that ELK3-mediated regulation of mitochondrial dynamics affected the chemotherapeutic efficacy of CDDP in TNBC cells. We showed that ELK3-suppressed TNBC cells were more sensitive to CDDP, and that this increase in sensitivity was caused by higher rates of mitochondrial fission, mitochondrial ROS production, and DNA damage. We further identified *DNM1L*, a gene encoding a major regulator of mitochondrial fission, the dynamin-related protein 1 (DRP1), as a direct downstream target of ELK3.

## Results

### *ELK3* knockdown enhances the CDDP sensitivity of TNBC cells

**Fig. 1:**
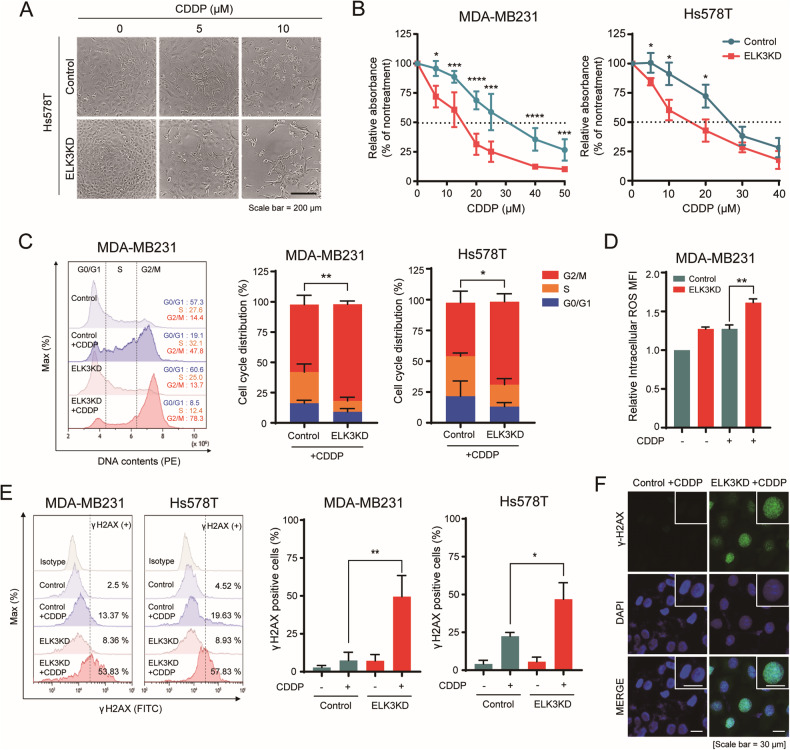
ELK3 depletion enhances the sensitivity of TNBC cells to CDDP. **A** Representative images of Hs578T control and ELK3KD-578T cells treated with the indicated concentrations of CDDP for 48 h. Scale bar, 200 µm. **B** Relative proportions of ELK3KD-TNBC cells and control cells after 48 h of treatment with the indicated CDDP concentrations. **C** Representative flow cytometry plot (left) and bar graph (right) showing the proportions of ELK3KD-231 and ELK3KD-578T cells in different phases of the cell cycle after incubation with CDDP (5 µM) for 48 h. Statistical analysis compared the number of control and ELK3KD-TNBC cells at G2/M phase. **D** Measurement of intracellular ROS levels in MDA-MB231 control and ELK3KD-231 cells in the presence or absence of CDDP. **E** Flow cytometric plots (left) and bar graphs (right) showing the proportions of γ-H2AX-positive ELK3KD-TNBC and control cells after treatment with CDDP (5 µM) for 48 h. **F** Immunocytochemical imaging of γ-H2AX in MDA-MB231 control and ELK3KD-231 cells after CDDP treatment. Scale bar, 30 µm. All experiments were repeated at least three times and performed in triplicate. *P* values were calculated using a two-tailed Student’s *t* test (**B**–**E**). Data represent the mean ± SD. *P < 0.05, **P < 0.01, ***P < 0.001, ****P < 0.0001.

Previously, we reported that the knockdown of *ELK3* with shRNA impaired autophagy and rendered MDA-MB231 cells more sensitive to the anticancer drug, doxorubicin [15]. Furthermore, other groups have reported that ELK3 is linked to platinum drug sensitivity in ovarian and breast cancer [16, 17]. Thus, we investigated whether the ELK3 expression levels in two representative human TNBC cell lines, MDA-MB231, and Hs578T, affected the response of these cells to CDDP. We previously generated the ELK3KD-578T and ELK3KD-231 cell lines, in which *ELK3* expression was knocked down, using an ELK3-targeting shRNA [13]. Treatment with various concentrations of CDDP for 48 h reduced the number of ELK3KD-578T and ELK3KD-231 cells to a greater extent than that of control cells (Fig. 1A, B). Additionally, the colony formation rate of ELK3KD-231 cells was significantly lower compared to the control cells upon treatment with CDDP (Supplementary Fig. 1). The anticancer effects of CDDP have been linked to cell cycle arrest because of its ability to crosslink the purine bases of DNA and inhibit DNA repair [18]. We therefore next assessed the effect of CDDP on the cell cycle of ELK3KD-TNBC cells. CDDP treatment halted ~1.5-fold more ELK3KD-231 and ELK3KD-578T cells in G2/M phase than control cells (Fig. 1C). Since the cytotoxic effects of CDDP are also related to the generation of intracellular ROS [19], we evaluated ROS levels in cancer cells following CDDP treatment. We found that the levels of intracellular ROS in ELK3KD-231 cells were much higher than those in control cells, irrespective of CDDP treatment (Fig. 1D). Consistent with the report that the accumulation of intracellular ROS can induce DNA damage and subsequent cell cycle arrest [20, 21], CDDP treatment resulted in a 1.5-fold greater increase in the number of γ-H2AX-positive ELK3KD-TNBC cells than in that of γ-H2AX-positive control cells (Fig. 1E, F). Taken together, these findings imply that *ELK3* knockdown rendered TNBC cells more sensitive to CDDP than control cells, by increasing ROS production and subsequently causing DNA damage and cell cycle arrest.

### CDDP induces mitochondrial ROS-dependent DNA damage in TNBC cells following *ELK3* knockdown

**Fig. 2:**
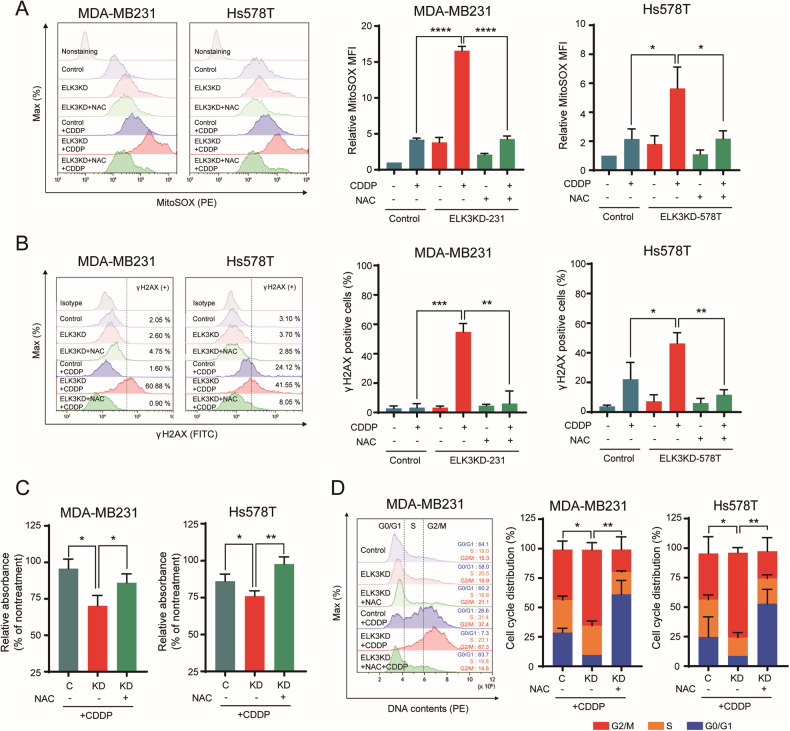
CDDP induces mitochondrial ROS-dependent DNA damage in ELK3-depleted TNBC cells. **A** Flow cytometry histograms (left) and bar graphs (right) showing the results of MitoSOX staining for mitochondrial ROS in the presence or absence of the ROS scavenger *N*-acetyl-l-cysteine (NAC) and CDDP (5 µM). **B** Flow cytometry histograms (left) and bar graphs (right) showing the proportions of γ-H2AX-positive ELK3KD-TNBC and control cells after treatment with different combinations of NAC and CDDP for 48 h. **C** Relative proportions of ELK3KD-TNBC and control cells treated with CDDP in the presence or absence of NAC for 48 h. **D** Representative flow cytometry histogram (left) and bar graphs (right) showing the proportions of ELK3KD-TNBC and control cells in different phases of the cycle after incubation with CDDP in the presence or absence of NAC for 48 h. Cell cycling was measured using propidium iodide staining, and statistical analysis was performed by comparing the numbers of cells in G2/M phase. All experiments were repeated three times and performed in triplicate. *P* values were calculated using a two-tailed Student’s *t* test (**A**–**D**). Data represent the mean ± SD. *P < 0.05, **P < 0.01, ***P < 0.001, ****P < 0.0001.

CDDP induces mitochondrial ROS accumulation in cancer cells, which contributes to genomic instability [22–24]. We therefore next investigated whether intracellular ROS in CDDP-treated ELK3KD-TNBC cells originated from mitochondria. We found that, after CDDP treatment, mitochondrial ROS levels were 3-fold higher in ELK3KD-TNBC cells than in control cells. Moreover, treatment with ROS scavengers (such as NAC and Trolox) restored mitochondrial ROS levels to those of the controls (Fig. 2A and Supplementary Fig. 2A). NAC (or Trolox) treatment also restored the number of γ-H2AX-positive ELK3KD-TNBC cells, induced by CDDP treatment, to that of controls (Fig. 2B and Supplementary Fig. 2B). Cotreatment of ELK3KD-TNBC cells with ROS scavengers (NAC or Trolox) and CDDP resulted in a considerable increase in the number of cells than when ELK3KD-TNBC cells were treated with CDDP alone (Fig. 2C and Supplementary Fig. 2C). Simultaneously, NAC (or Trolox) cotreatment significantly lowered the number of CDDP-treated ELK3KD-TNBC cells arrested in G2/M phase (Fig. 2D and Supplementary Fig. 2D). Together, these findings led us to conclude that excessive mitochondrial ROS accumulation was the primary cause of ELK3KD-TNBC cell chemosensitivity to CDDP.

### CDDP accelerates mitochondrial fission and mitochondrial ROS production in ELK3-depleted TNBC cells

**Fig. 3:**
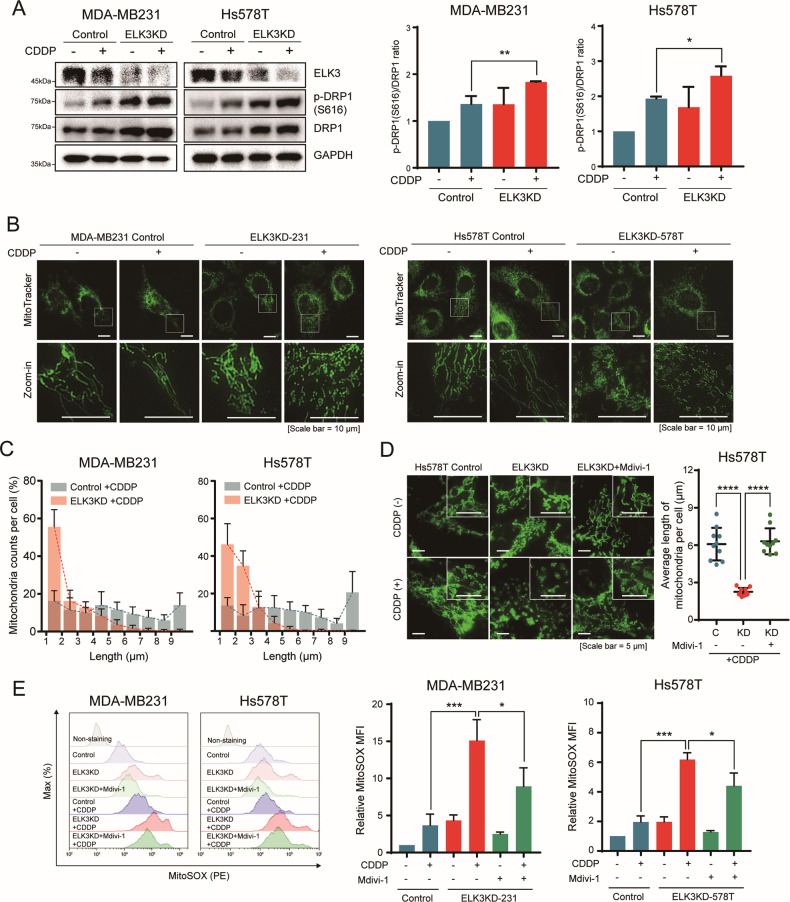
CDDP accelerates mitochondrial fission and mitochondrial ROS production in ELK3-depleted TNBC cells. **A** Immunoblot (left) showing amounts of total and phosphorylated (Ser616) DRP1 in CDDP-treated ELK3KD-TNBC and control cells. GAPDH was used as a loading control. Bar graphs (right) showing the p-DRP1 (S616) per DRP1 ratio of the immunoblot, which was quantified using ImageJ software. **B** Imaging of mitochondrial morphology of ELK3KD-TNBC and control cells in the presence or absence of CDDP for 48 h, using MitoTracker staining. Scale bar, 10 µm. **C** Bar graph showing the proportions of mitochondria of different lengths (quantified using ImageJ) per cell, averaged over a total of 10 cells per group. **D** Imaging of mitochondrial morphology using MitoTracker staining (left) in Hs578T control, ELK3KD-578T, and ELK3KD-578T cells treated with a mitochondria division inhibitor (Mdivi-1) in the presence or absence of CDDP. Scale bar, 5 µm. The average length of mitochondria per cell was measured in ten cells from each CDDP-treated group using ImageJ (right). **E** Flow cytometry histograms (left) and bar graphs (right) showing the results of MitoSOX staining of mitochondrial ROS in ELK3KD-TNBC and control cells after treatment with different combinations of Mdivi-1 and CDDP. All experiments were performed at least three times and in triplicate. *P* values were calculated using a two-tailed Student’s *t* test (**A**, **D**, and **E**). Data represent the mean ± SD. *P < 0.05, **P < 0.01, ***P < 0.001, ****P < 0.0001; n.s. not significant.

We previously demonstrated that inhibiting ELK3 expression in TNBC cells shifted mitochondrial dynamics toward fission [14]. Several studies have reported that mitochondrial fission increases the chemosensitivity of various cancer cell types to CDDP [25–29]. Hence, we next questioned whether CDDP accelerated ELK3KD-dependent mitochondrial fission in TNBC cells. We found that CDDP induces phosphorylation of DRP1 in both ELK3KD-TNBC and control cells. Phosphorylated DRP1 is recruited to the outer mitochondrial membrane and stimulates mitochondrial fission [30]. We found that the amount of phosphorylated (on Ser616) DRP1 in CDDP-treated ELK3KD-TNBC cells was higher than that in control cells (Fig. 3A). Consistently, ELK3KD-TNBC cells exhibited increased mitochondrial fission following CDDP treatment compared to controls (Fig. 3B, C). These results indicate that the mitochondria of ELK3KD-TNBC cells were more susceptible to undergoing a fission transition following CDDP treatment than those of control TNBC cells with wild-type ELK3 levels. Given that mitochondrial fission enhances ROS production [31, 32], we anticipated that the overproduction of mitochondrial ROS in CDDP-treated ELK3KD-TNBC cells could be reversed by blocking mitochondrial fission. As expected, cotreatment of ELK3KD-TNBC cells with CDDP and Mdivi-1 (a pharmacological inhibitor of mitochondrial fission) markedly reduced mitochondrial ROS levels (Fig. 3D, E). Together, these findings suggest that CDDP promoted mitochondrial fission in ELK3KD-TNBC cells, which accelerated mitochondrial remodeling and induced excessive mitochondrial ROS production.

### CDDP-mediated cell cycle arrest and DNA damage in ELK3-depleted TNBC cells are associated with mitochondrial fission

**Fig. 4:**
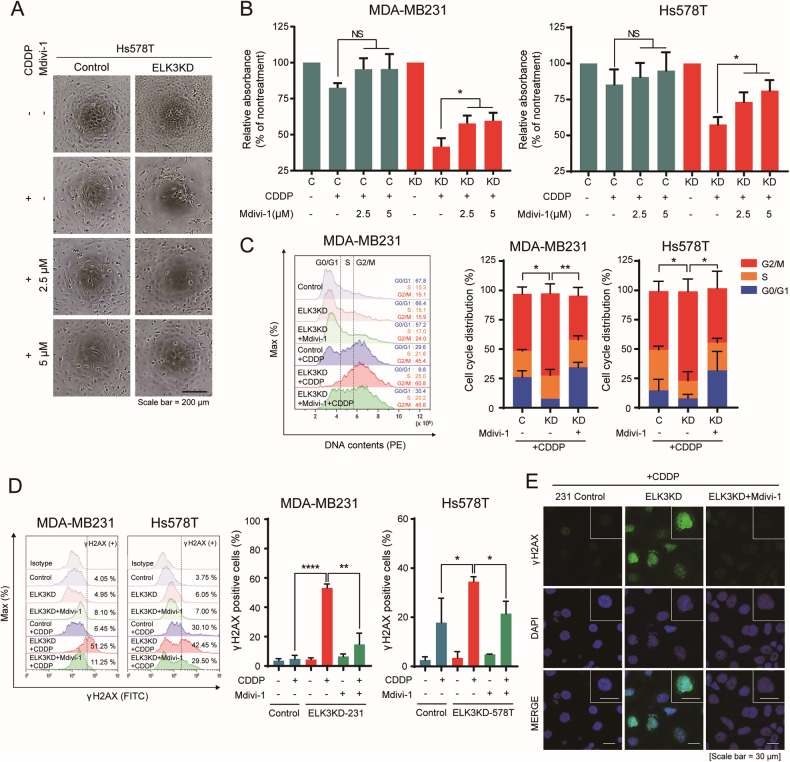
CDDP induces DNA damage and cell cycle arrest in ELK3-depleted TNBC cells by accelerating mitochondrial fission. **A** Representative images showing Hs578T control and ELK3KD-578T cells treated with different combinations of 5 µM CDDP and Mdivi-1 for 48 h. Scale bar, 200 µm. **B** Bar graphs showing the proportions of cells after treatment with different combinations of 5 µM CDDP and Mdivi-1 for 48 h. **C** Representative flow cytometry histogram (left) and bar graphs (right) showing the proportions of ELK3KD-TNBC and control cells in different phases of the cell cycle after treatment with different combinations of CDDP and Mdivi-1 for 48 h. The cell cycle was analyzed using propidium iodide staining, and statistical analysis was performed by comparing the proportions of cells in G2/M phase. **D** Flow cytometry histograms (left) and bar graphs (right) showing the proportions of γ-H2AX-positive ELK3KD-TNBC and control cells after treatment with different combinations of Mdivi-1 and CDDP for 48 h. **E** Immunocytochemical imaging of γ-H2AX in MDA-MB231 control and ELK3KD-231 cells after CDDP treatment in the presence or absence of Mdivi-1. Scale bar, 30 µm. All assays were repeated at least three times and performed in triplicate. *P* values were calculated using a two-tailed Student’s *t* test (**B**–**D**). Data represent the mean ± SD. *P < 0.05, **P < 0.01, ****P < 0.0001; n.s. not significant.

Given the above results and a recent report that mitochondrial fission promotes DNA damage [33], we next questioned whether CDDP-induced excessive mitochondrial fission and mitochondrial ROS production in ELK3KD-TNBC cells contributed to both cell cycle arrest and CDDP sensitivity. We observed that the number of CDDP-treated ELK3KD-TNBC cells was increased by the pharmacological inhibition of mitochondrial fission (Fig. 4A, B). In addition, the increase in the number of G2/M-arrested and γ-H2AX-positive ELK3KD-TNBC cells (induced by CDDP treatment) was significantly reversed following Mdivi-1 administration (Fig. 4C–E). In conclusion, CDDP-induced excessive mitochondrial fission in ELK3KD-TNBC cells, which caused them to arrest in G2/M phase.

### ELK3 functions as a transcriptional repressor of *DNM1L*, and the ELK3-DRP1 axis determines CDDP sensitivity in TNBC cells

**Fig. 5:**
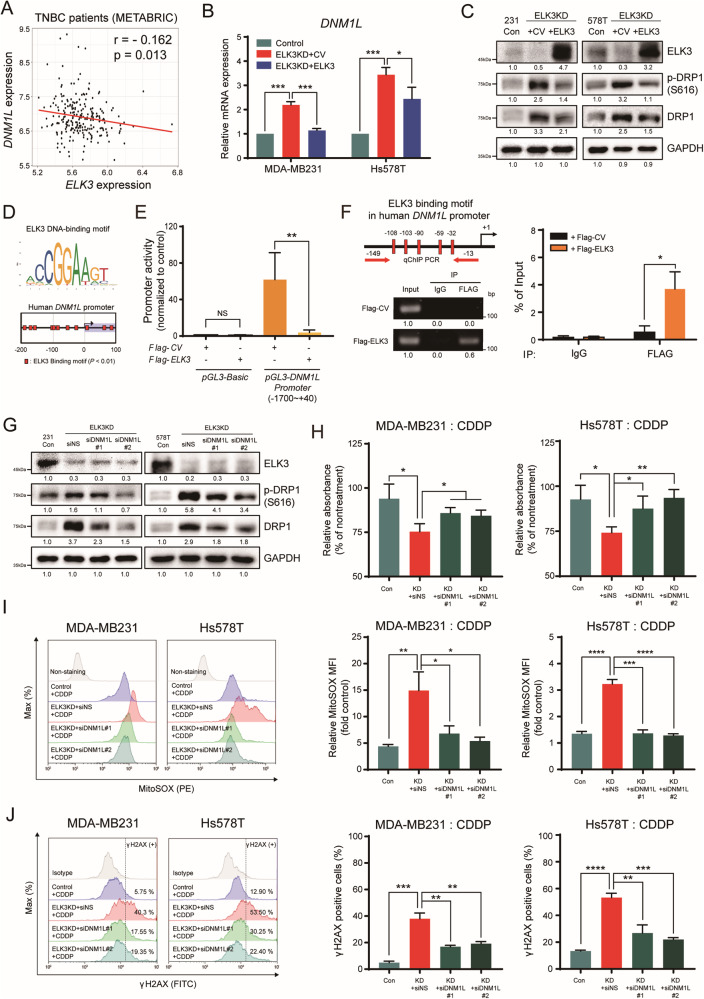
ELK3 is transcriptional repressor of *DNM1L* and the ELK3-DRP1 axis determines CDDP sensitivity in TNBC cells. **A** Correlation between *ELK3* and *DNM1L* expression levels in 236 TNBC patients. Expressional datasets were obtained from the METABRIC database. **B** Quantitative real-time PCR analysis of *ELK3* and *DNM1L* expression in ELK3KD-TNBC and control cells transfected with a control plasmid or an ELK3-expressing plasmid. **C** Immunoblot analysis of total and phosphorylated (Ser616) DRP1 in control and ELK3KD-TNBC cells transfected with a control plasmid or an ELK3-expressing plasmid. Band intensities on the immunoblot were measured using ImageJ, and the results are displayed beneath the blot. **D** ELK3 binding motif (shown as red boxes) analysis in the *DNM1L* promoter. ELK3 binding motif alignment was performed using the Eukaryotic Promoter Database (*P* value < 0.01). **E** The luciferase assay was performed using the pGL3 reporter plasmid harboring the *DNM1L* promoter region (−1.70–0.04 kb). The luciferase reporter plasmid was cotransfected with the control plasmid (Flag-CV) or the ELK3-expressing plasmid (Flag-ELK3) into ELK3KD-231 cells, as indicated. **F** ChIP-qPCR analysis of ELK3 binding to the *DNM1L* promoter. ELK3KD-231 cells were transfected with the Flag-ELK3-expressing plasmid. The genomic DNA was immunoprecipitated with an anti-Flag antibody and subjected to qPCR using primers specific for the *DNM1L* promoter region (−149 to −13 bp). **G** Immunoblot analysis of total and phosphorylated (Ser616) DRP1 in control and ELK3KD-TNBC cells transfected with nonspecific (siNS) or *DNM1L*-targeting siRNA (si*DNM1L*). Band intensities on the immunoblot were measured using ImageJ, and the results are displayed beneath the blot. **H** Relative proportions of control and ELK3KD-TNBC cells transfected with siNS or si*DNM1L* after treatment with 5 µM CDDP for 48 h. **I** Measurement of mitochondrial ROS levels and **J** γ-H2AX-positive control and ELK3KD-TNBC cells transfected with a siNS or si*DNM1L* after treatment with CDDP for 48 h. All experiments were repeated at least three times and performed in triplicate. *P* values were calculated using a two-tailed Student’s *t* test (**B**, **E**, **F**, **H**–**J**) and Pearson’s correlation coefficient (**A**). Data represent the mean ± SD. *P < 0.05, **P < 0.01, ***P < 0.001, ****P < 0.0001; n.s. not significant.

**Fig. 6:**
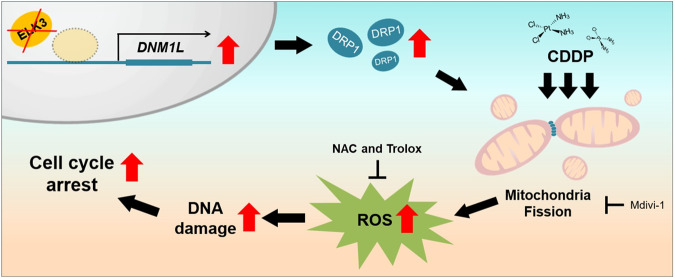
Scheme of the effect of ELK3-DRP1 axis on the chemosensitivity of CDDP in TNBC cells.

We next set out to identify target genes that govern mitochondrial dynamics, the expression of which is suppressed by the transcriptional activity of ELK3. Figure 3A and Supplementary Fig. 4 show that, in the absence of CDDP, DRP1 was overexpressed in ELK3KD-TNBC cells. To examine whether there was a correlation between *ELK3* and *DNM1L* (encodes DRP1) expression, we performed a bioinformatics analysis of *ELK3* and *DNM1L* expression patterns in TNBC patients using data from the METABRIC. As shown in Fig. 5A, *DNM1L* expression was weakly but significantly negatively correlated with *ELK3* expression. We next subdivided TNBC patients into two group according to *ELK3* expression level (*ELK3*^Low^ group or *ELK3*^High^ group), and then examined the correlation of each group with *DNM1L* expression. Our findings showed that *ELK3*^High^ group has lower expression of *DNM1L* compared to the *ELK3*^Low^ group in TNBC patients (Supplementary Fig. 3). Quantitative real-time PCR and immunoblotting results indicated that *DNM1L* expression was elevated in ELK3KD-TNBC cells but decreased when ELK3 expression was restored (Fig. 5B, C). As shown in Fig. 5D, multiple ELK3 binding motifs were enriched near the transcriptional initiation site (+1) of the *DNM1L* promoter. To verify whether ELK3 repressed *DNM1L* promoter activity, we conducted a promoter luciferase assay using a cloned *DNM1L* promoter (−1.70–0.04 kb). We showed that the promoter activity of *DNM1L* in ELK3KD-231 cells was significantly repressed by the ectopic expression of ELK3 (Fig. 5E). Also, the ChIP-qPCR results revealed that the ectopically expressed ELK3 bound directly to the *DNM1L* promoter in ELK3KD-231 cells (Fig. 5F). These results suggest that ELK3 functioned as a transcriptional repressor of *DNM1L* and directly repressed its expression in TNBC cells. Lastly, we evaluated the effect of a *DNM1L*-targeting siRNA (si*DNM1L*) on the CDDP sensitivity of ELK3KD-TNBC cells. In the presence of CDDP, the number of si*DNM1L*-transfected ELK3KD TNBCs was considerably greater than that of ELK3KD-TNBC cells transfected with the control siRNA (siNS) (Fig. 5G, H). As predicted, silencing of DRP1 leads to the restoration of shorter mitochondria length in ELK3KD-231 cells in the presence of CDDP (Supplementary Fig. 5). Thus, the inhibition of DRP1 expression in ELK3KD-TNBC cells substantially diminished the excessive production of mitochondrial ROS and the accumulation of γ-H2AX-positive cells in response to CDDP treatment (Fig. 5I, J). Taken together, these results demonstrate that the ELK3-DRP1 axis determined CDDP sensitivity in TNBC by modulating mitochondrial dynamics and ROS accumulation (Fig. 6).

## Discussion

Although CDDP is a commonly used and effective chemotherapeutic agent, the intrinsic or acquired resistance of cancer cells restricts its efficacy. Understanding the underlying mechanism that determines the sensitivity of cancer cells to CDDP may thus enable the development of more effective therapeutic strategies.

Previously, we reported that ELK3 increases the sensitivity of TNBC cells to the cytotoxicity of natural killer cells by regulating cancer cell mitochondrial dynamics [14]. In the present study, we demonstrated that the biological significance of ELK3-mediated modulation of mitochondrial dynamics could be extended to the chemotherapy of TNBC cells. Specifically, we found that ELK3-depleted TNBC cells were more susceptible to the effects of CDDP. The fact that the pharmacological suppression of mitochondrial fission and the scavenging of mitochondrial ROS could reduce the chemosensitivity of ELK3KD-TNBC cells to CDDP implies that the CDDP-mediated acceleration of mitochondrial fission is the primary cause of this chemosensitivity. It is therefore reasonable to assume that control TNBC cells with normal ELK3 expression might exhibit some resistance to CDDP by suppressing mitochondrial fission. By contrast, ELK3KD-TNBC cells, which lack the transcriptional repressor (ELK3) required to suppress the expression of mitochondrial fission genes, succumbed to CDDP-induced damage.

In ovarian cancer, chemoresistance is acquired and maintained through distal superenhancer-driven transcriptional reprogramming [17]. ELK3, the expression of which is considerably elevated in CDDP-resistant ovarian cancers, was identified as one of these superenhancer-associated transcription factors [17]. Combined with our results, this suggests that the ability of ELK3 to induce chemoresistance in cancer cells might be conserved in TNBC and ovarian cancer cells. Moreover, ELK3 could be a valuable therapeutic target for improving the efficacy of CDDP-mediated chemotherapy. Although in this study we focused primarily on the role of ELK3 in regulating mitochondrial dynamics to explain the chemosensitivity of TNBC cells to CDDP, we do not rule out the possibility that ELK3 also employs alternative mechanisms to regulate the response of cancer cells to chemotherapy. For example, we have previously shown that ELK3KD-231 cells exhibit defective autophagy, which is directly related to their chemosensitivity to doxorubicin [15]. Since CDDP-induced autophagy protects breast cancer cells against the effects of chemotherapy [34, 35], it remains to be seen whether the impaired autophagy of ELK3KD-TNBC cells also contributed to the chemosensitivity of these cells to CDDP.

It is critical to understand how accelerated mitochondrial fission and the overproduction of mitochondrial ROS are associated with the DNA damage and cell cycle arrest incurred by CDDP-treated ELK3KD-TNBC cells. As shown by the accumulation of γ-H2AX, a sensitive marker for DNA double-strand breaks, CDDP severely damages the DNA of ELK3KD-TNBC cells (Fig. 1E).

Unlike ionizing radiation or xenotoxic agents such as doxorubicin, etoposide, and tirapazamine [36], CDDP does not induce direct double-strand breaks. However, mitochondrial fission promotes DNA damage via the sublethal activation of caspases [37]. Hence, we questioned whether the acceleration of mitochondrial fission by CDDP-induced caspase-mediated DNA breaks. However, the use of caspase inhibitors in combination with CDDP did not reduce the chemosensitivity of ELK3KD-TNBC cells (data not shown), indicating that the CDDP-mediated DNA damage experienced by ELK3KD-TNBC cells was independent of caspase activity. Given that CDDP treatment can cause interstrand DNA crosslinking and subsequent replication fork damage, which leads to double-strand breaks [38], one possible explanation for the substantial accumulation of γ-H2AX following CDDP treatment is that ELK3KD-TNBC cells are more likely to experience replication stress.

Since the coadministration of a ROS scavenger and CDDP significantly reduced the number of γ-H2AX-positive and cell cycle-arrested ELK3KD-TNBC cells, we speculate that the overproduction of mitochondrial ROS was the main cause of γ-H2AX accumulation. In general, ROS induces DNA damage by oxidizing nucleoside bases, which are then typically recognized and repaired by the base excision repair pathway [39]. Also, the oxidation of dNTPs by ROS affects polymerase activity and replication fork progression [40]. Therefore, it is reasonable to speculate that CDDP could induce double-strand breaks in ELK3KD-TNBC cells via two mechanisms: (1) by directly promoting the interstrand crosslinking of genomic DNA and (2) by indirectly increasing ROS-mediated oxidative DNA damage. It has also been reported that the proliferation of ELK3KD-TNBC cells is 1.5-fold higher than that of control cells [13]. Given that increased speeds of replication fork progression induce DNA replication stress [41], the higher rates of proliferation experienced by ELK3KD-TNBC cells may additionally render these cells more prone to CDDP-induced damage.

We identified *DNM1L*, which encodes a key regulator of mitochondrial fission, DRP1, as a direct downstream target of ELK3. Combined with our previous report that ELK3 directly represses the expression of *MiD51* [14], which encodes a DRP1 adapter protein, we speculate that ELK3 regulates mitochondrial dynamics, at least in TNBC cells, by repressing multiple major mitochondrial fission genes.

High levels of mitochondrial fission gene expression correlate with reduced breast cancer metastasis and improved survival outcomes in TNBC patients [42]. Thus, as a transcriptional repressor of mitochondrial fission-inducing genes, we predict that ELK3 could potentially be targeted to sensitize TNBC cells to chemotherapy.

## Methods

### Cell culture and knockdown systems

MDA-MB231 and Hs578T were purchased from the American Type Culture Collection (Manassas, VA, USA). Dulbecco’s modified Eagle medium (DMEM; Gibco, Grand Island, NY, USA) supplemented with 10% fetal bovine serum (FBS; Gibco) and 1% penicillin–streptomycin (Gibco) was used to culture MDA-MB231 cells. Hs578T cells were cultured in DMEM supplemented with 10% FBS, 1% penicillin–streptomycin, and 0.01 mg/mL insulin (Sigma, St. Louis, MO, USA). Stable knockdown of *ELK3* in MDA-MB231 and Hs578T cells was achieved using retroviral vectors expressing short hairpin (sh)RNA targeting ELK3, as described previously [13]. All cells were incubated at 37 °C, 5% CO_2_.

### Transfection

Cancer cells were seeded in 100 mm dishes (5 × 10^5^ cells/dish) and transfected with small interfering (si)RNAs or plasmids in the presence of Lipofectamine 2000 (Invitrogen, Carlsbad, CA, USA), according to the manufacturer’s protocols. Supplementary Table 1 lists the plasmids and siRNAs used in this study.

### MTT assay

To measure cell viability, 2500 cells were seeded in a 96-well plate and treated with CDDP (Dong-A Parm, Seoul, South Korea) at the indicated concentrations for 48 h. 5 mg/mL 2,5-diphenyl-2H-tetrazolium bromide (MTT) solution (Sigma) was then added to each well, followed by a 2 h incubation at 37 °C. The medium was then replaced with 200 µl of dimethyl sulfoxide, and the plates were analyzed on a microplate reader (Molecular Devices, San Jose, CA, USA) to determine the absorbance of each well. The following formula was used to calculate: relative absorbance (%) = (absorbance of the CDDP-treated group/absorbance of the CDDP-nontreated group) × 100.

### Chemicals

The ROS scavenger, *N*-acetyl cysteine (NAC), was purchased from Sigma (Cat. No. A7250) and used at a concentration of 5 mM. Mdivi-1, a small mitochondrial division inhibitor, was purchased from Sigma (Cat. No. M0199) and used at a concentration of 5 µM.

### Cell cycle analysis

Cells were harvested as a single-cell suspension and fixed in 70% ethanol. Fixed cells were washed with cold phosphate-buffered saline (PBS) and stained with propidium iodide (Invitrogen) solution at a final concentration of 50 µg/mL in the presence of 0.2 mg/mL RNase A (Bioneer, Daejeon, Republic of Korea) and 0.1% Triton X-100 (Sigma). Cell cycling was then evaluated on a CytoFLEX flow cytometer (Beckman Coulter, Indianapolis, IN, USA).

### Flow cytometry analysis

Cells were harvested as a single-cell suspension and fixed in 4% paraformaldehyde. Cells were washed with Dulbecco’s (D)PBS (Gibco) and then permeabilized with FACS buffer (BD Biosciences, San Jose, CA, USA) containing 0.1% Trition X-100 for 30 min. After incubation for 2 h at room temperature with a primary antibody, cells were stained with a fluorochrome-conjugated secondary antibody. The cells were acquired on a CytoFLEX flow cytometer. Supplementary Table 2 lists the antibodies in this study.

### Detection of ROS

Total ROS levels were evaluated using the Cellular ROS Assay Kit (Abcam, Cambridge, UK) by following the manufacturer’s manual. To detect mitochondrial ROS, cells were incubated for 20 min at 37 °C in a medium containing 5 µM MitoSOX (Invitrogen) and then washed twice with DPBS. Fluorescence was measured using a CytoFLEX flow cytometer.

### Immunoblotting

Cells were lysed with RIPA buffer (Cell signaling, Danvers, MA, USA) containing a protease/phosphatase inhibitor cocktail (Thermo Fisher Scientific, Rochester, NY, USA) for 30 min. Total protein was extracted and heated for 10 min at 95 °C. Proteins were then separated on sodium dodecyl sulfate-polyacrylamide gels and transferred to polyvinylidene difluoride membranes (Bio-Rad, Hercules, CA, USA). Membranes were stained with the indicated antibodies, and immunoreactivity was detected using the enhanced chemiluminescence solution (Thermo Fisher Scientific). Supplementary Table 2 lists the antibodies used for immunoblotting.

### Quantitative chromatin immunoprecipitation PCR (ChIP-qPCR)

Cells were fixed in a 1% paraformaldehyde solution and incubated at room temperature for 15 min to crosslink proteins and genomic (g)DNA. Glycine was added to fixed cells at a final concentration of 125 mM to quench the formaldehyde. Cells were washed with PBS and lysed with cell lysis buffer (Cell Signaling) containing protease/phosphatase inhibitors. Cell lysates were sonicated on ice to fragment gDNA and then centrifuged at 15,000 × *g* for 15 min at 4 °C. The supernatants were incubated overnight at 4 °C with antibodies (anti-Flag or IgG), prior to the addition of protein A/G magnetic beads (Thermo Fisher Scientific). The complexes were washed with RIPA buffer. SDS and proteinase K were added to separate immunoprecipitated DNA from DNA-protein complexes. Prior to use in ChIP-qPCR, the immunoprecipitated DNA was purified with phenol/chloroform. The amount of precipitated chromatin was calculated as a percentage of the input sample. Supplementary Table 3 lists the primers used in this study.

### Mitochondrial imaging

Cells were seeded in a 96-well black/clear bottom plate (2500 cells/well). To stain mitochondria, cells were incubated for 30 min at 37 °C with 1 µM of MitoTracker Green (Invitrogen) and then washed with DPBS. Mitochondria were photographed using an EVOS microscope (Thermo Fisher Scientific).

### Promoter luciferase assay

*ELK3* knockdowned MDA-MB231 cells were transfected with the control plasmid or the ELK3-expressing plasmid in the presence of *Renilla*-expressing plasmids by Lipofectamine 2000. 48 h later, the transfected cells were lysed with cell lysis buffer. The Dual-Luciferase Reporter Assay Kit (Promega, Madison, WI, USA) was used to measure luciferase activity. The firefly luciferase activity values were normalized against *Renilla* luciferase activity values.

### Bioinformatics analysis of public breast cancer datasets

Molecular Taxonomy of Breast Cancer International Consortium (METABRIC) breast cancer datasets was downloaded from cBioPortal on 1 September 2021 [43]. Patients were screened by selecting cases with ‘negative’ estrogen receptor status, ‘negative’ progesterone receptor status, a ‘claudin-low, basal’ PAM50-subtype, and a cancer type described as ‘breast invasive ductal carcinoma.’ 236 TNBC cases were identified by combining clinical data with gene expression data.

### Statistical analysis

GraphPad Prism v.7.0 software was used for statistical analysis (GraphPad Software, La Jolla, CA, USA). An unpaired Student’s *t* test was used to determine the statistical significance of differences. Graphical data were presented as the mean ± standard deviation (SD), obtained from at least three independent experiments, using three replicates per sample. Pearson’s correlation analysis was used to evaluate the correlation between *ELK3* and *DNM1L* expression. *P* values < 0.05 were considered as a measure of statistical significance.

## Supplementary information

Supplementary information

Agreement

## Data Availability

Data are available from the corresponding author upon reasonable request.
