# Supplementary material for: The ELK3-DRP1 axis determines the chemosensitivity of triple-negative breast cancer cells to CDDP by regulating mitochondrial dynamics
Source: Cell Death Discov. 2023 Jul 8;9:237. doi: 10.1038/s41420-023-01536-5 (PMC10329652; doi:10.1038/s41420-023-01536-5)
Supplement: Supplementary file 1 — Supplementary information [file 41420_2023_1536_MOESM1_ESM.docx]

Supplementary information for **The ELK3-DRP1 axis determines the chemosensitivity of triple negative breast cancer cells to CDDP by regulating mitochondrial dynamics**

Joo Dong Park^1^, Hye Jung Jang^1^, Seung Hee Choi^1^, Gae Hoon Jo^1^, Jin-Ho Choi^1^,

Sohyun Hwang^1^, Wooram Park^2^, Kyung-Soon Park^1^*

1 Department of Biomedical Science, CHA University, Seongnam, Republic of Korea

2 Department of Integrative Biotechnology, Sungkyunkwan University, Suwon, Republic of Korea

* Correspondence to: Kyung-Soon Park, kspark@cha.ac.kr

**Supplementary Table 1. Plasmids and small interfering (si)RNAs used in the study**

| No | Name | Additional information |
| --- | --- | --- |
| 1 | pLenti-cMyc-DDK | Origene, PS1000064 |
| 2 | pLenti-ELK3-cMyc-DDK | NM_005230.3 |
| 3 | pRL-TK | Promega, E2231 |
| 4 | pGL3-Basic | Promega, E1751 |
| 5 | pGL3-hDNM1L promoter (−1.70 to 0.04 kb) | - |
| 6 | Nonspecific siRNA control | Bioneer, SN-1003 |
| 7 | hDNM1L siRNA | Bioneer, 10059 |

**Supplementary Table 2. Antibodies used in the study**

| Antibody | Manufacturer/supplier | Catalog No. | Application |
| --- | --- | --- | --- |
| ELK3 | Novus Biologicals | NBP2-01264 | Immunoblot |
| p-DRP1(S616) | Cell Signaling | 3455S | Immunoblot |
| DRP1 | Cell Signaling | 5391T | Immunoblot |
| GAPDH | Santa Cruz Biotechnology | sc-166574 | Immunoblot |
| Flag | MBL International Corporation | M185-3L | ChIP |
| γ-H2AX | Abcam | ab22551 | Flow cytometry |

Abbreviations: ChIP, chromatin immunoprecipitation.

**Supplementary Table 3. Primers used in the study**

| Genes | Forward Primer (5' to 3') | Reverse Primer (5' to 3') | Application |
| --- | --- | --- | --- |
| *ELK3* | ACC CAA AGG CTT GGA AAT CT | TGT ATG CTG GAG AGC AGT GG | qRT-PCR |
| *DNM1L* | CCA AGG TGC CTG TAG GTG AT | CAG CAG TGA CAG CGA GGA TA | qRT-PCR |
| *GAPDH* | GGG TGT GAA CCA TGA GAA | GTC TTC TGG GTG GCA GTG AT | qRT-PCR |
| *DNM1L Promoter (−149 to −13 bp)* | CAG GCC TTG CTC CTC TCC A | GGC CCA CAG TTC GCC TCC | ChIP-qPCR |
| *DNM1L Promoter (−1.70 to 0.04 kb)* | ATG CTA GCC CTC TCC TCA CCT GCT TTA ATT | ATC TCG AGT CTG AAA ACA CGG GGC C | Luciferase assay |

Abbreviations: ChIP, chromatin immunoprecipitation; qPCR, quantitative polymerase chain reaction.


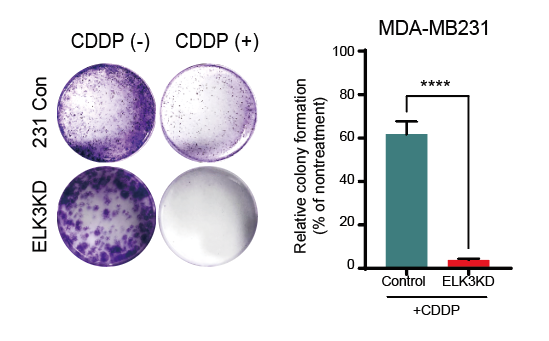


**Supplementary Figure 1. The effect of ELK3 expression level on the capacity of MDA-MB231 cells to form colonies under conditions of prolonged CDDP treatment** (Left) Representative image and (Right) a quantified bar graph displaying the colony formation rate of MDA-MB231 control cells (231 Con) and ELK3KD-231 cells (ELK3KD) following treatment with 5 µM of CDDP for a week. *P*-values were calculated using a two-tailed Student’s t-test. Data represent the mean ± SD. *****P < 0.0001*

**
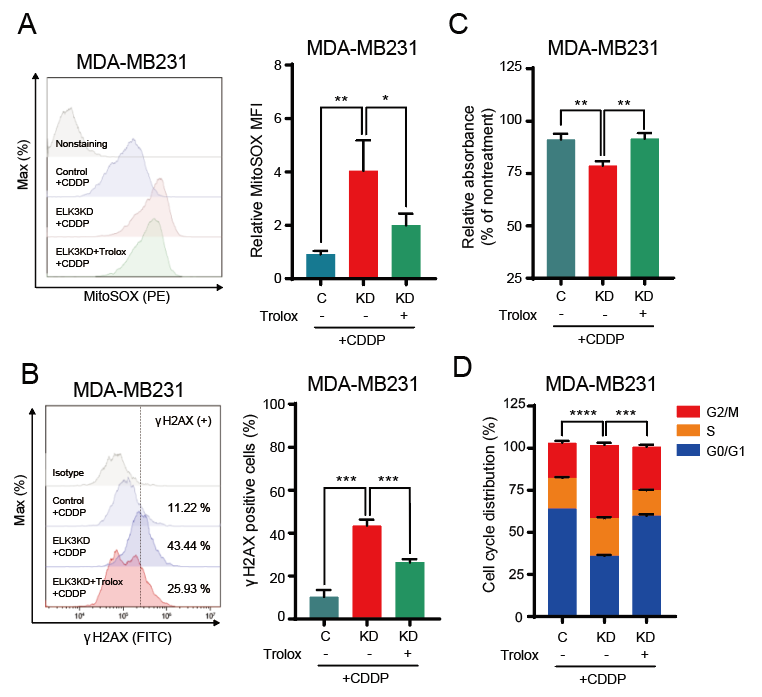
**

**Supplementary Figure 2. Effect of Trolox on CDDP sensitivity of ELK3-depleted MDA-MB231 cells.** (A) (left) Flow cytometry histogram and (right) corresponding bar graph of MitoSOX staining in MDA-MB231 control and ELK3KD-231 cells after treatment with 5 µM CDDP for 48 h, in the presence or absence of 50 µM Trolox, a ROS scavenger. (B) (left) Flow cytometric histogram and (right) the quantified data of γ-H2AX-positive cells in MDA-MB231 control and ELK3KD-231 cells after treatment with CDDP, in the presence or absence of Trolox. (C) Relative proportions of MDA-MB231 and ELK3KD-231 cells treated with CDDP, in the presence or absence of Trolox. (D) Cell cycle analysis of MDA-MB231 control and ELK3KD-231 cells after CDDP treatment, in the presence or absence of Trolox. Statistical analysis was performed by comparing the percent of cells in the G2/M phase. *P*-values were calculated using a two-tailed Student’s t-test (A–D). Data represent the mean ± SD. **P < 0.05*, ***P < 0.01,* ****P <0.001,* *****P < 0.0001*.

**
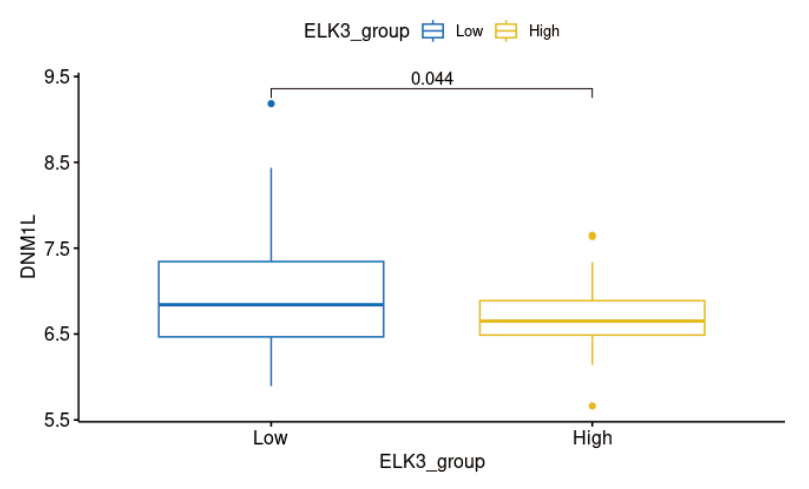
**

**Supplementary Figure 3. *DNM1L* expression level according to *ELK3* expression level in METABRIC TNBC patients.** TNBC patients with *ELK3* expression in the bottom 15 % were labeled as “*ELK3*^low^” (n=36). Conversely, TNBC patients with *ELK3* expression in the top 15% were labeled as “*ELK3*^high^” (n=36). *P*-values were calculated using a Student’s t-test. Data represent the mean ± SD.

**
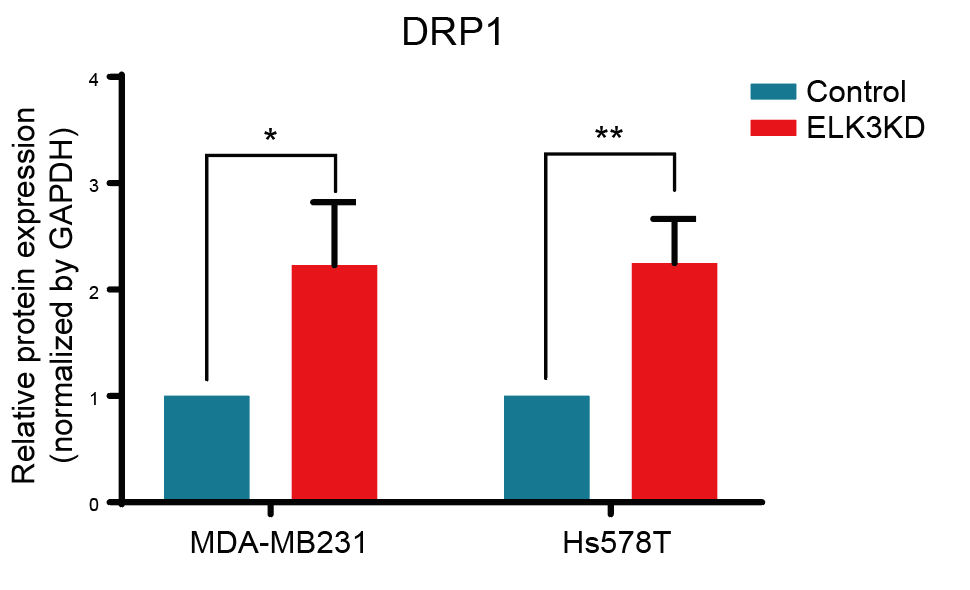
**

**Supplementary Figure 4. DRP1 protein expression level in ELK3KD-TNBC cells.** Immublot results of DRP1 expression level in Figure 3A were quantified by ImageJ software. *P*-values were calculated using a two-tailed Student’s t-test. Data represent the mean ± SD. **P < 0.05*, ***P < 0.01*.

**
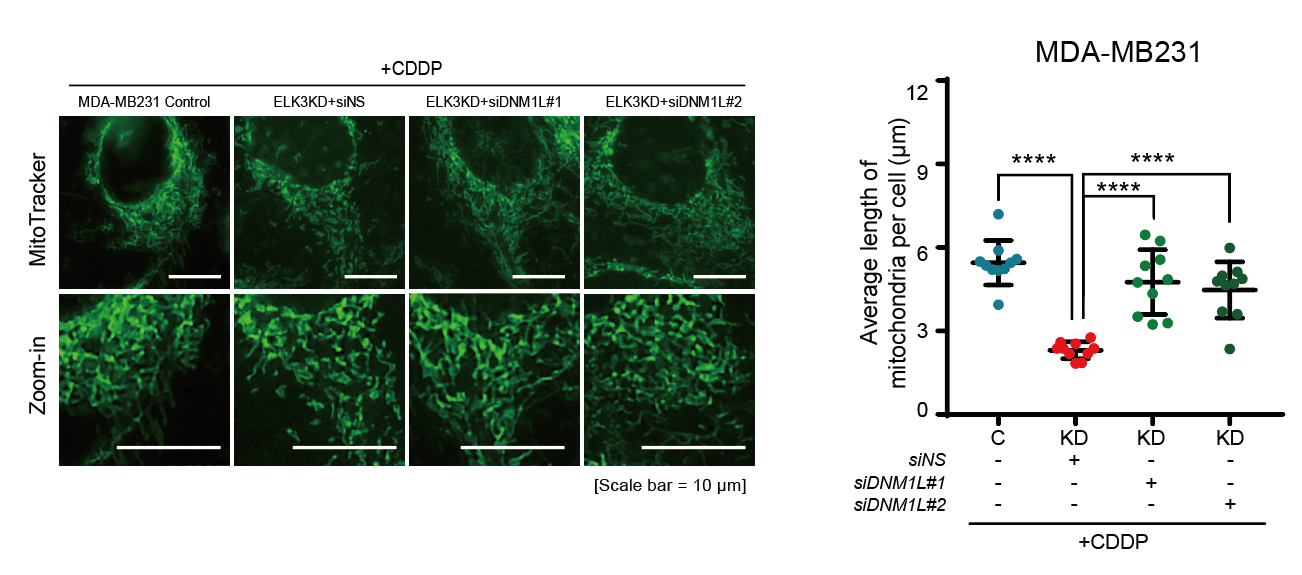
**

**Supplementary Figure 5 The effect of siRNA-mediated *DNM1L* suppression on the mitochondrial length of ELK3KD-231 cells in the presence of CDDP.** (left) Representative imaging results are presented on the left side, showing MitoTracker staining of mitochondria in MDA-MB231 control, ELK3KD-231, and ELK3KD-231 cells transfected with two siRNAs targeting *DNM1L*. Scale bar, 10 µm. The quantified data is displayed as a bar graph on the right side, indicating the average length of mitochondria per cell. The measurements were performed on 10 cells using ImageJ software. *P*-values were determined using a two-tailed Student’s t-test to assess statistical significance. Data represent the mean ± SD. *****P < 0.0001*.
