# Supplementary material for: The ELK3-DRP1 axis determines the chemosensitivity of triple-negative breast cancer cells to CDDP by regulating mitochondrial dynamics
Source: Cell Death Discov. 2023 Jul 8;9:237. doi: 10.1038/s41420-023-01536-5 (PMC10329652; doi:10.1038/s41420-023-01536-5)
Supplement: Supplementary file 2 — Agreement [file 41420_2023_1536_MOESM2_ESM.docx]

Dear editor

Thank you very much for sharing the news of our manuscript's acceptance to *Cell Death Discovery*. Below is an email to the other co-authors, asking for their consent to include Professor Wooram Park, as you requested.

Best regards,

Joo Dong Park
The first author of the manuscript

| Authors | |
| --- | --- |
| Joo Dong Park | O |
| Hye Jung Jang | O |
| Seung Hee Choi | O |
| Gae Goon Jo | O |
| Jin-Ho Choi | O |
| Sohyun Hwang | O |
| Wooram Park | O |
| Kyung-Soon Park | O |

-----Original Message-----
**From:** "박주동"<wnehd06@naver.com>
**To:** "장혜정"<ccc1931@naver.com>; "최승희"<raru1201@naver.com>; "조계훈"<hoon1866@naver.com>; "최진호"<harryho0903@naver.com>; "황소현"<blissfulwin@chamc.co.kr>; "박경순"<kspark@cha.ac.kr>;
**Cc:** "Wooram Park"<parkwr@skku.edu>;
**Sent:** 2023-06-23 (금) 12:05:44 (GMT+09:00)
**Subject:** [논문승인] FW: Fwd: Decision letter for CDDISCOVERY-23-0720R: Accepted in Principle

Dear Colleagues,

I am delighted to inform you that our manuscript CDDISCOVERY-[23-0720](tel:230720)R, entitled "The ELK3-DRP1 Axis Determines the Chemosensitivity of Triple Negative Breast Cancer Cells to CDDP by Regulating Mitochondrial Dynamics," has been provisionally accepted for publication in Cell Death Discovery.

I would like to express my gratitude to the colleagues who have assisted us.

During the revision process, Professor Wooram Park engaged in discussions regarding the manuscript's results.

We have duly acknowledged his contribution and included him as an author of the paper.

If you concur with this decision, kindly respond to us via email.

Sincerely,

Joo Dong Park

First author of the manuscript

**Ms. Hye Jung Jang**

-----Original Message-----
**From:** "장혜정"<ccc1931@naver.com>
**To:** "박주동"<wnehd06@naver.com>;
**Cc:** "박경순교수님"<kspark@cha.ac.kr>; "최승희"<raru1201@naver.com>; <harryho0903@naver.com>;
**Sent:** 2023-06-23 (금) 13:14:18 (GMT+09:00)
**Subject:** RE: [논문승인] FW: Fwd: Decision letter for CDDISCOVERY-23-0720R: Accepted in Principle

Dear Joo Dong Park,

I wholeheartedly agree with the decision to include Professor Wooram Park as an author.

Congratulations on the acceptance of our manuscript!

 Well done and thank you for all your hard work!

Warmest regards,

Hye Jung. Jang

장혜정. Hye Jung. Jang

Bio medical science, CHA univ.

Tel. 010. 4946. 1931

E-mail. ccc1931@naver.com, jung183779 @gmail.com

**Ms. Seung Hee Choi**

-----Original Message-----
**From:** "최승희"<raru1201@naver.com>
**To:** "박주동"<wnehd06@naver.com>;
**Cc:** "박경순 교수님"<kspark@cha.ac.kr>; "장혜정"<ccc1931@naver.com>;
**Sent:** 2023-06-23 (금) 12:17:43 (GMT+09:00)
**Subject:** RE: [논문승인] FW: Fwd: Decision letter for CDDISCOVERY-23-0720R: Accepted in Principle

Dear Joo Dong Park,

I hope this email finds you well.

I am pleased to inform you that I wholeheartedly concur with the decision to include Professor Wooram Park as an author of the paper.

I am writing to express my sincere congratulations on the final acceptance of our manuscript, "The ELK3-DRP1 Axis Determines the Chemosensitivity of Triple Negative Breast Cancer Cells to CDDP by Regulating Mitochondrial Dynamics," for publication in Cell Death Discovery.

I would like to personally express my appreciation for yours valuable contributions.

Once again, congratulations to everyone involved in this achievement.

Warmest regards,

Seung Hee Choi

Bio medical science, CHA univ.
Tel. 010. 8770. 0930
E-mail. raru1201@naver.com, choicat14@gmail.com

**Mr. Gae Hoon Jo**

-----Original Message-----
**From:** "조계훈"<hoon1866@naver.com>
**To:** "박주동"<wnehd06@naver.com>;
**Cc:**
**Sent:** 2023-06-23 (금) 12:27:17 (GMT+09:00)
**Subject:** RE: [논문승인] FW: Fwd: Decision letter for CDDISCOVERY-23-0720R: Accepted in Principle

Deer Mr. Park

I agree with Professor Wooram Park's contribution and inclusion as an author.

Best Regards,

GaeHoon Jo

**Mr. Jin-Ho Choi**

-----Original Message-----
**From:** "최진호"<harryho0903@naver.com>
**To:** "박주동"<wnehd06@naver.com>;
**Cc:**
**Sent:** 2023-06-23 (금) 12:25:51 (GMT+09:00)
**Subject:** RE: [논문승인] FW: Fwd: Decision letter for CDDISCOVERY-23-0720R: Accepted in Principle

Dear Park,

I'm glad to hear that our manuscript has been accepted.

And I also agree with acknowledgement of Professor Wooram Park.

Thank you

**Professor. Sohyun Hwng**

-----Original Message-----
**From:** "황소현"<blissfulwin@chamc.co.kr>
**To:** "박경순"<kspark@cha.ac.kr>; "박주동"<wnehd06@naver.com>; "장혜정"<ccc1931@naver.com>; "최승희"<raru1201@naver.com>; "조계훈"<hoon1866@naver.com>; "최진호"<harryho0903@naver.com>;
**Cc:** "Wooram Park"<parkwr@skku.edu>;
**Sent:** 2023-06-23 (금) 13:50:03 (GMT+09:00)
**Subject:** Re: Re: [논문승인] FW: Fwd: Decision letter for CDDISCOVERY-23-0720R: Accepted in Principle

Dear Joo Dong Park,

Congratulation! And thank you for the good news.

Regarding including Prof. Wooram Park as an author of the paper, I agree with your decision. =)

Best wishes,

Sohyun Hwang

Associate Professor

Department of Pathology, CHA University School of Medicine

CHA Bundang Medical Center

Department of Biomedical Sciences, CHA University

Tel: 031-780-6019

E-mail: blissfulwin@cha.ac.kr

Special Issue "Frontiers in Genetics and Genomics of Ovarian Cancer"

<https://www.mdpi.com/journal/genes/special_issues/genes_ovarian_cancer>

**Professor. Wooram Park**

-----Original Message-----
**From:** "Wooram Park"<parkwr@skku.edu>
**To:** <kspark@cha.ac.kr>;
**Cc:** <wnehd06@naver.com>; <ccc1931@naver.com>; <raru1201@naver.com>; <hoon1866@naver.com>; <harryho0903@naver.com>; <blissfulwin@chamc.co.kr>;
**Sent:** 2023-06-24 (토) 23:52:31 (GMT+09:00)
**Subject:** Re : Re: [논문승인] FW: Fwd: Decision letter for CDDISCOVERY-23-0720R: Accepted in Principle

Dear Kyung-Soon Park and Colleagues,

I am writing to express my sincere congratulations on the provisional acceptance of your manuscript, "**The ELK3-DRP1 Axis Determines the Chemosensitivity of Triple Negative Breast Cancer Cells to CDDP by Regulating Mitochondrial Dynamics**" for publication in ***Cell Death Discovery***. The work you and your team have done on this research is commendable, and its impact on the field will be significant.

I also want to express my heartfelt gratitude for including me as an author in this paper. It was a privilege to be part of the discussions during the revision process and contribute to the development of the results. I greatly appreciate the acknowledgment of my contributions.

I am pleased to concur with your decision to include me as an author and look forward to seeing the final publication of our manuscript.

Once again, congratulations on this achievement and thank you for including me in this significant work.

Kind Regards,

Wooram

**Wooram Park, Ph.D.,**

Assistant Professor,

Department of Integrative Biotechnology,

College of Biotechnology and Bioengineering,

Sungkyunkwan University (SKKU),

2066 Seobu-ro, Jangan-gu, Suwon, Gyeonggi 16419, Republic of Korea

Office: +82-31-299-4857

Fax: +82-31-290-7870

Cell: +82-10-5720-7610

E-mail: parkwr@skku.edu

Web-site: [https://wparklab.weebly.com](https://wparklab.weebly.com/)

**Professor. Kyung-Soon Park**

-----Original Message-----
**From:** "박경순"<kspark@cha.ac.kr>
**To:** "박주동"<wnehd06@naver.com>; "장혜정"<ccc1931@naver.com>; "최승희"<raru1201@naver.com>; "조계훈"<hoon1866@naver.com>; "최진호"<harryho0903@naver.com>; "황소현"<blissfulwin@chamc.co.kr>;
**Cc:** "Wooram Park"<parkwr@skku.edu>;
**Sent:** 2023-06-23 (금) 12:27:58 (GMT+09:00)
**Subject:** Re: [논문승인] FW: Fwd: Decision letter for CDDISCOVERY-23-0720R: Accepted in Principle

Dear Joo Dong Park,

I agree with the decision to include Professor Wooram Park as an co-author of the paper.

Sincerely yours,

Kyung-Soon Park
